# Integrate thermostabilized fusion protein apocytochrome *b*_*562*_RIL and N-glycosylation mutations: A novel approach to heterologous expression of human UDP-glucuronosyltransferase (UGT) 2B7

**DOI:** 10.3389/fphar.2022.965038

**Published:** 2022-08-12

**Authors:** Jia Xue, Haitao Zhang, Su Zeng

**Affiliations:** ^1^ Zhejiang Province Key Laboratory of Anti-Cancer Drug Research, Institute of Drug Metabolism and Pharmaceutical Analysis, College of Pharmaceutical Sciences, Zhejiang University, Hangzhou, Zhejiang, China; ^2^ Zhejiang Province Key Laboratory of Anti-Cancer Drug Research, Hangzhou Institute of Innovative Medicine, Institute of Pharmacology and Toxicology, College of Pharmaceutical Sciences, Zhejiang University, Hangzhou, Zhejiang, China; ^3^ The Second Affiliated Hospital, Zhejiang University School of Medicine, Hangzhou, Zhejiang, China

**Keywords:** UDP-glucuronosyltransferase 2B7, expression and purification, thermostabilization, mutations, enzymatic activity, surface plasmon resonance, transmission electron microscopy

## Abstract

Human UDP-glucuronosyltransferase (UGT) 2B7 is a crucial phase II metabolic enzyme that transfers glucuronic acid from UDP-glucuronic acid (UDPGA) to endobiotic and xenobiotic substrates. Biophysical and biochemical investigations of UGT2B7 are hampered by the challenge of the integral membrane protein purification. This study focused on the expression and purification of recombinant UGT2B7 by optimizing the insertion sites for the thermostabilized fusion protein apocytochrome *b*_562_RIL (BRIL) and various mutations to improve the protein yields and homogeneity. Preparation of the recombinant proteins with high purity accelerated the measurement of pharmacokinetic parameters of UGT2B7. The dissociation constants (*K*_D_) of two classical substrates (zidovudine and androsterone) and two inhibitors (schisanhenol and hesperetin) of UGT2B7 were determined using the surface plasmon resonance spectroscopy (SPR) for the first time. Using negative-staining transmission electron microscopy (TEM), UGT2B7 protein particles were characterized, which could be useful for further exploring its three-dimensional structure. The methods described in this study could be broadly applied to other UGTs and are expected to provide the basis for the exploration of metabolic enzyme kinetics, the mechanisms of drug metabolisms and drug interactions, changes in pharmacokinetics, and pharmacodynamics studies *in vitro.*

## 1 Introduction

**FIGURE 1:**
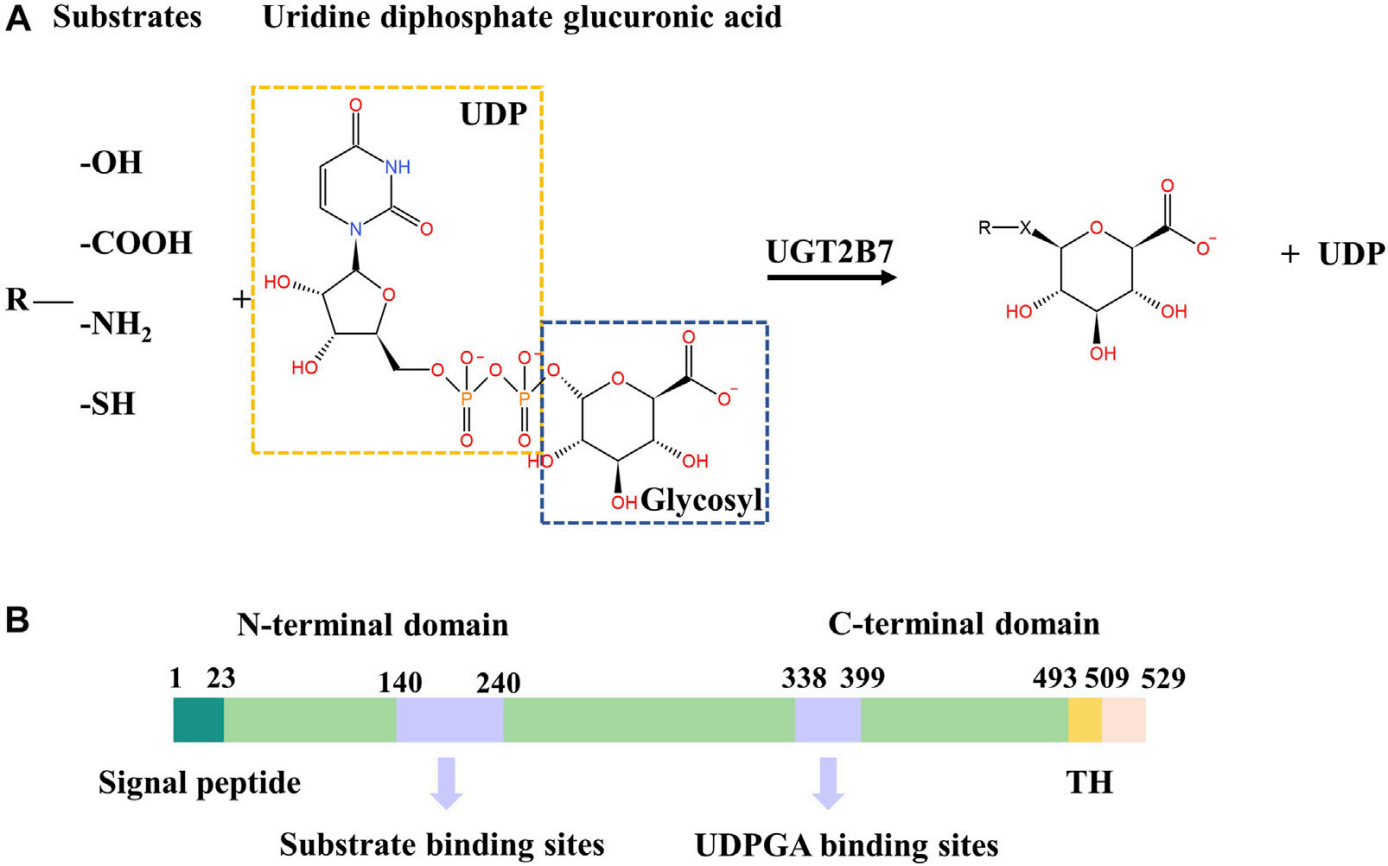
**(A)** Conjugation reaction catalyzed by UGT2B7. X represents -O^-^, -COO^-^, -NH^+^, -S^-^. **(B)** Color-coded domain architecture of human UGT2B7. The numbers represent the residue positions of the wide-type UGT2B7 counted from the N-terminus. The locations of the substrate and UDPGA binding sites are indicated in purple. TH, transmembrane helix.

The human UGT2B7 is a major phase II metabolism enzyme that detoxifies a large amount of essential endobiotic and xenobiotics (Meech and MacKenzie, 1998) (Rowland et al., 2013) (Tukey and Strassburg, 2000), such as morphine (Ofoegbu and Ettienne, 2021), zidovudine (AZT) (Uchaipichat et al., 2008), estriol (Sneitz et al., 2013), hyodeoxycholic acid (HDAC) (Bock, 2012), and androsterone (Gall et al., 1999), by catalyzing the transfer of the glucuronic acid group from uridine diphosphate glucuronic acid (UDPGA) to a specific substrate (also known as aglycone). The major nucleophilic chemical groups of specific substrates include hydroxyl, carboxyl, amino, and sulfhydryl (Figure 1A). UGT2B7 plays an essential role in the endobiotic homeostasis and metabolic defense systems, but its enzymatic activity can be inhibited by the natural compounds such as schisanhenol (Song et al., 2015), licochalcone A (LAC) (Xin et al., 2016), and hesperetin (Liu et al., 2016). Moreover, some drugs, including shikonin (Cheng et al., 2019), emodin (Wu et al., 2018), cannabinoids, and their metabolites (Nasrin et al., 2021), act as inhibitors of UGT2B7 which have a high-risk of toxicity due to the drug-drug interactions. In addition, some environmental pollutants, such as polycyclic aromatic hydrocarbons (PAHs) (Yang et al., 2020) and bromophenols (BPs) (Wang et al., 2020) can also inhibit the enzymatic activity of UGT2B7.

The human UGTs are classic type Ⅰ transmembrane glycoproteins, mainly localized in the endoplasmic reticulum (ER) membrane, consisting of approximately 530 amino acids (Radominska-Pandya et al., 2002). UGTs have two domains, the N-terminal domain (NTD) with a cleavable signal peptide accountable for targeting the ER membrane and the C-terminal transmembrane domain (CTD) anchoring the protein to the ER membrane. At the CTD, a single transmembrane helix (TH) contains about 20 residues that transmits the phospholipid bilayer from lumen to the cytosol (Magdalou et al., 2010) (Figure 1B). The flexible single transmembrane helix makes the three-dimensional structures of full-length human UGTs still undetermined, which limits the elucidation of the molecular mechanisms for UGTs. However, two CTDs structures of UGT2B7 and UGT2B15 have been solved, while the substrates and UDPGA binding sites are still elusive (Miley et al., 2007) (Zhang et al., 2020).

The rapid progress of surface plasmon resonance (SPR) spectroscopy is beneficial for studying the ligand-binding to proteins (O’connor et al., 2022) (Speck et al., 2022), which enables the quantitative and real-time measurements of the binding affinity and kinetics of protein-ligand interactions using relatively small amounts of materials, at medium-throughput (Mizuguchi et al., 2012) (Drozd et al., 2021) (Yin et al., 2020). The dissociation constant (*K*_D_) is commonly obtained using SPR spectroscopy for ligand-binding screening, but few studies have been illustrated for UGTs.

In this study, we developed a novel method to express the full-length human UGT2B7 using the Bac-to-Bac baculovirus expression system, by introducing the thermostabilized fusion protein apocytochrome *b*_*562*_RIL (BRIL), which is widely used in the purification of membrane proteins such as G protein-coupled receptors (GPCRs) to improve the protein yields and homogeneity (Xiang et al., 2016) (Liu et al., 2012). In addition, site-directed mutagenesis has been used to increase the protein expression levels and thermostability (Zhang et al., 2017), or remove the post-translational modifications (PTMs) not critical for the protein function (Zhang et al., 2012a). We examined the thermostability and enzyme kinetics of the recombinant UGT2B7 proteins. Moreover, we characterized the protein particles using the negative-stain TEM, and determined the ligand-binding affinity of two classical substrates (zidovudine and androsterone) and two inhibitors (schisanhenol and hesperetin) using SPR. Our method could be applied to other UGTs with improved yields and purity, and is expected to facilitate the substrate screening studies and three-dimensional structure determination, for the pharmacokinetics and pharmacodynamics research on UGTs enzymes.

## 2 Materials and methods

### 2.1 Chemicals and reagents

Zidovudine, UDPGA, dimethylsulfoxide (DMSO), and alamethicin were purchased from Sigma Chemical Co. (St. Louis, MO, United States). Androsterone, schisanhenol, hesperetin, and UDP were purchased from MedChemExpress (State of New Jersey, United States). Zidovudine O-glucuronide was purchased from Toronto Research Chemicals (Toronto, Canada). Aristolochic acid A (AAI) was purchased from the National Institutes for Food and Drug Control (Beijing, China). High-purity acetonitrile for high-performance liquid chromatography (HPLC) coupled with MS/MS was obtained from Merck (Darmstadt, Germany).

### 2.2 Constructs design and protein expression

The full-length human UGT2B7 cDNA was cloned into the pFastBac1 vector with thermostabilized fusion protein apocytochrome *b*_562_RIL (BRIL) followed by a 10 × His-tag at the C-terminus using restriction endonuclease *Bam*H Ⅰ and *Xho* Ⅰ (Supplementary Figure S1). Two truncations at the N terminus (M1-L10 and M1-C23) and 17 mutations were introduced into UGT2B7 using standard QuickChange PCR.

The plasmids were transformed into *Escherichia coli* DH10Bac cells, and then the bacmids were transfected into *Spodoptera frugiperda* (*Sf*9) insect cells. The Bac-to-Bac baculovirus expression system was used to generate high-titer recombinant baculovirus, and the virus was amplified from P0 to P1. The *Sf*9 cells were grown to a density of 2.1 × 10^6^ cells/mL in ESF921 serum-free media and infected with the 100 folds volume of P1 viruses. After 48 h post-infection at 27°C, the cells were collected by centrifugation and cell pellets were stored at −80°C.

### 2.3 Protein purification

Cell pellets were isolated by repeated dounce homogenization twice in the hypotonic buffer [10 mM HEPES (pH 7.5), 20 mM KCl, 10 mM MgCl_2,_ and protease inhibitor cocktail (Rocha)] and the hypertonic buffer [10 mM HEPES (pH 7.5), 1.0 M NaCl, 20 mM KCl, 10 mM MgCl_2_ and protease inhibitor cocktail (Rocha)]. Cell membranes were collected by centrifugation at 58,000 × g for 30 min. The purified membranes were resuspended in a buffer containing 10 mM HEPES (pH 7.5), 20 mM KCl, 10 mM MgCl_2_, 2 mg/ml iodoacetamide (Sigma-Aldrich) and incubated at 4°C for 30 min. After incubation, the membranes were solubilized in 50 mM HEPES (pH 7.5), 800 mM NaCl, 20% (v/v) glycerol, 20 mM imidazole, 0.5% lauryl maltose neopentyl glycol (LMNG, Anatrace), 0.05% cholesteryl hemisuccinate tris salt (CHS, Anatrace) at 4°C for 4 h.

Insoluble materials were removed by centrifugation at 58,000 × g for 1 h and the supernatant was incubated with pre-equilibrated Talon IMAC resin (TaKaRa) overnight at 4°C. Then the resin was packed into a gravity column (Bio-Rad) and washed with 20 column volumes wash buffer 1 of 50 mM HEPES (pH 7.5), 400 mM NaCl, 5% (v/v) glycerol, 0.1% LMNG, 0.01% CHS, 30 mM imidazole, and 20 column volumes wash buffer 2 of 20 mM HEPES (pH 7.5), 200 mM NaCl, 5% (v/v) glycerol, 0.01% LMNG, 0.001% CHS, 45 mM imidazole. The protein was eluted in 10 column volumes elution buffer of 20 mM HEPES (pH 7.5), 150 mM NaCl, 0.002% LMNG, 0.0002% CHS, 250 mM imidazole. Eluted UGT2B7 was concentrated at about 0.5 mg/ml using Vivaspin Turbo Ultrafiltration Units (MWCO 50 kDa). The final yields of the purified complexes were ∼0.4 mg/L of insect cell culture. Elution buffer was exchanged into desalt buffer consisting of 20 mM HEPES (pH 7.5), 150 mM NaCl, 0.002% LMNG, and 0.0002% CHS using the HP desalting column (GE Healthcare). The pooled samples were further analyzed by SDS polyacrylamide gel electrophoresis (SDS-PAGE) and stained with Coomassie blue.

To compare the homogeneity between different constructs, 10 μl concentrated protein was centrifuged at 4 °C, 13,400 g for 10min, and the supernatant was analyzed using an Agilent 1260 HPLC (Agilent Technologies, Santa Clara, CA, United States). The proteins were analyzed on a Sepax Nanofilm SEC-250 column (4.6 mm × 300 mm, 5 μm, 250 Å), in the desalt buffer at a flow rate of 0.5 ml/min, and monitored by UV absorbance at 280 nm.

### 2.4 Negative stain transmission electron microscopy

Before checking samples using TEM, the eluted protein was concentrated to 500 μL and subjected to size exclusion chromatography on a Superdex 200 Increase 10/300 column (GE Healthcare) preequilibrated in desalt buffer. Peak fractions containing monomers of UGT2B7 were pooled and concentrated to ∼0.5 mg/ml. Then samples collected were diluted to 0.001 mg/ml with desalting buffer. 2.5 μl sample was spotted on a freshly glow-discharged 300 mesh carbon grid, stained with 2% uranyl-acetate for three times, and imaged on Tecnai G2 spirit 120 kV electron microscopy (Thermo FEI) at 180,000 × magnification.

### 2.5 Kinetic analyses

Cell pellets were isolated by dounce homogenization and incubated in a buffer containing 100 mM potassium phosphate (pH 7.4), 10 mM MgCl_2_, 0.05 mg/ml alamethicin, 1 mg/ml UGT2B7, zidovudine (concentration in the range of 0–5 mM), and 5 mM UDPGA to a total volume of 50 μl. After a 5 min preincubation at 37°C, the reaction was started by adding UDPGA to a final concentration of 5 mM, continued at 37°C for 120 min, and then was terminated by adding 100 μl ice-cold acetonitrile with Aristolochic acid A as the internal standard (final concentration of 100 ng/ml) to precipitate the protein. After centrifugation at 4°C, 12,000 g for 10 min, the supernatant was analyzed by liquid chromatography coupled with mass spectrometry (LC-MS/MS). Concentrations of AZTG in cell lysates were determined using an Agilent 1290/6460 LC-MS (Agilent Technologies, Santa Clara, CA, United States) with a triple quadrupole mass spectrometer. The separation was analyzed on a ZORBAX XDB column (50 mm × 2.1, 3.5 μm) eluted with a gradient mobile phase consisting of 0.1% formic acid-water and 0.1% formic acid-acetonitrile at a flow rate of 0.2 ml/min. The mass spectrometric analysis was performed using an electron spray ionization (ESI) source in negative ion mode and the ion pair AZTG at m/z 442.3 → 125.0, and AAI in positive ion mode at m/z 342.1 → 296.0 (Divi et al., 2008) (Badée et al., 2019). For quantitation, the peak area ratio of analyte to internal standard was compared with ratios obtained from a standard curve containing known amounts of analyte.

The software GraphPad Prism, version 8.0 (GraphPad Software Inc., San Diego, USA) was employed for kinetic analysis. The equations, including the Michaelis-Menten equation,

$$V=\frac{V_{\text{max}}\times S}{K_{\text{m}}+S}$$

was used for the kinetic data calculation, where *V* is the velocity of the reaction, *V*_max_ is the maximum velocity, *S* is the substrate concentration, and *K*_*m*_ is the Michaelis-Menten constant.

### 2.6 Thermostability assay

To assess the thermostability of the solubilized UGT2B7, 2–3 μg purified protein was incubated in a volume of 120 μl at 4°C for 15 min in the presence of 1 μM 7-diethylamino-3-(4-maleimidophenyl)-4-methylcoumarin (CPM, Sigma-Aldrich), DMSO (0.1% final concentration, v/v), 25 mM HEPES (pH 7.5), 500 mM NaCl, 10% (v/v) glycerol, 0.002% (w/v) LMNG, 0.0002% (w/v) CHS. After incubation of the sample at 4°C for 15 min, the thermostability of UGT2B7 was monitored using Cary Eclipse Fluorescence Spectrophotometer (Agilent Technologies, Santa Clara, CA, United States) with temperature ramping from 20°C to 95°C in 1°C steps at wavelengths of 384 nm (excitation) and 463 nm (emission). The gain setting was determined at the beginning of the run and the melting temperature (T_m_) was calculated from the point of inflection.

### 2.7 Surface plasmon resonance

Binding affinities of different substrates or inhibitors to the purified UGT2B7 proteins were analyzed using SPR at 25°C on a Biacore T200 with a CM7 sensor chip (GE Healthcare). All experiments were performed with a running buffer containing phosphate buffer (pH 7.4), 0.002% LMNG, and 0.0002% CHS to maintain the concentration of detergent above the critical micelle concentration, which is necessary for the formation of micelles and the correct folding of the purified UGT2B7 proteins. About 8,500 response units of 6 × His-tag monoclonal antibody (Proteintech) were directly immobilized on the chip, which were desalted in 10 mM sodium acetate (pH 4.5). Then the purified UGT2B7 proteins flowed over the chip and coupled with the His-tag antibody at about 18,000 RU. And the blank channel was used as the negative control. Substrates or inhibitors solutions flowed over the chip at a flow rate of 30 μl/min and were contacted 120 s. Then, flowed running buffer at the same rate to dissociation 120 s. The obtained affinities data were analyzed with the BIA evaluation software using the steady-state affinity model.

## 3 Results

### 3.1 Expression and purification

#### 3.1.1 Optimization of *b*_562_RIL insertion sites

**FIGURE 2:**
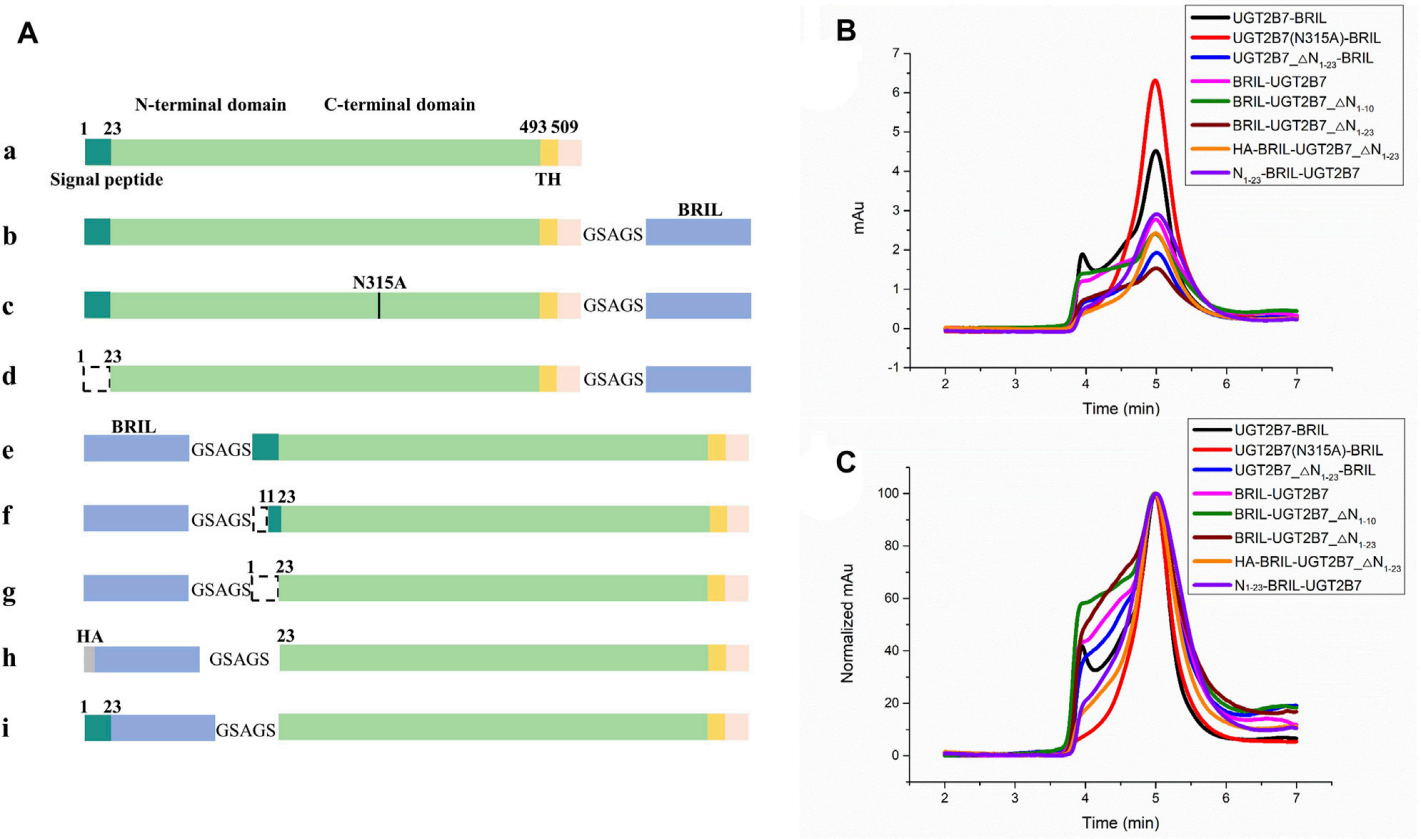
Screening for insertion sites of the fusion protein BRIL to UGT2B7. **(A)** Color-coded domain architecture of different insertion sites. The dashed line represents the truncation. HA, hemagglutinin signal peptide. **(B)** Size exclusion chromatography curves of different constructs at a flow rate of 0.5 ml/min. The *X*-axis represents the time and *Y*-axis shows the UV absorption at 280 nm representing the yields of protein. Fused BRIL at the C-terminus increased the yields of the full-length UGT2B7 (blank curve). Fused BRIL at the N-terminus, regardless of the signal peptide length, the proteins were not properly expressed and the aggregation was significantly high (magenta, olive, and wine curves). The addition of the haemagglutinin (HA) signal peptide or the supplementation of the UGT2B7 signal peptide (residues N1-23) before BRIL (orange and purple curves) showed better homogeneity and higher yields. **(C)** The alignment of all curves in B shows the protein homogeneity.

It is suggested that deletions of the C-terminal transmembrane helix (TH) and di-lysine motif (DM, residues 524–529) have no impact on the UGT2B7 location in ER (Miyauchi et al., 2019). Besides, the CTDs of UGTs are conserved, and the fused His-tag to the UGTs C-terminus have no impact on the glucuronidation activity (Zhang et al., 2012b). Our results showed that fusion of BRIL at the C-terminus of UGT2B7 with a 5-residue linker could significantly increase the yields of full-length UGT2B7, but with high aggregation (Figures 2A,B. The blank curve in Figures 2B,C).

The signal peptide (residues N1-23) at the N-terminus of UGT2B7 plays a key role in targeting ER by binding to the signal recognition particle (Ouzzine et al., 1999). By truncating the signal peptide, the protein yields were reduced and the aggregation was increased (Figures 2A,d. The blue curve in Figures 2B,C). Fusion of BRIL at the N-terminus, regardless of the signal peptide length, the UGT2B7 proteins were not properly expressed and the aggregation was significantly high (Figure 2A,e,f,g. The magenta, olive, and wine curves in Figures 2B,C). However, addition of the haemagglutinin (HA) signal peptide or the supplementation of the UGT2B7 signal peptide (residues N1-23) before BRIL (Figure 2A,h,i. The orange and violet curves in Figures 2B,C) showed better homogeneity and higher yields (Supplementary Figures S3A,C). It has been reported that most of the amino acids (residues 1-493) of UGT2B7 are located at the luminal side with variable conformations (Nair et al., 2015). We speculated that when BRIL was fused at the N-terminus inside ER, it might be detrimental to the protein expression. Furthermore, the signal peptide sequence of UGT2B7 is essential for the protein to recognize the ER membrane.

#### 3.1.2 Optimization of mutations

**FIGURE 3:**
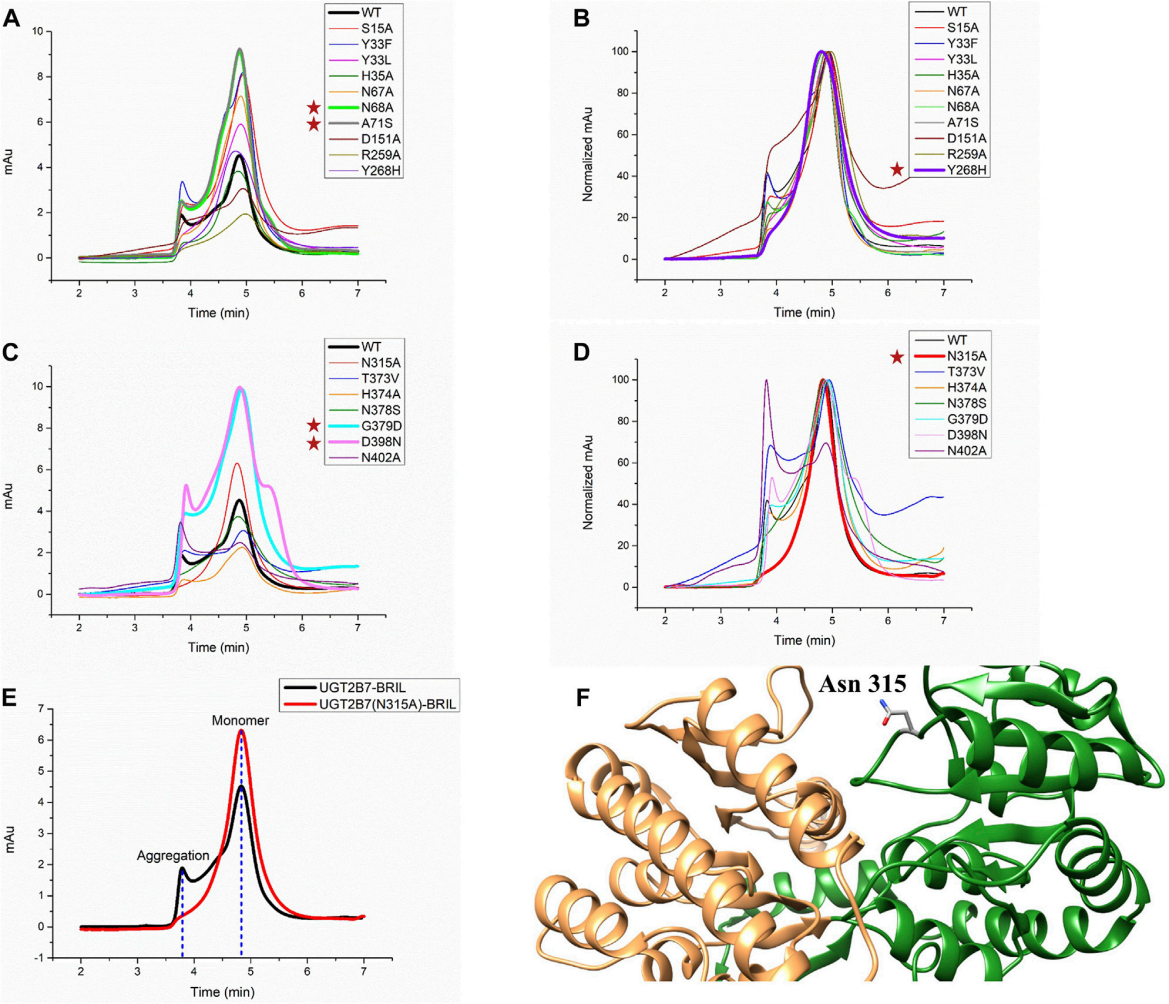
Screening for mutations of UGT2B7-BRIL. **(A)** Diagram of size exclusion chromatography of different mutations at the UGT2B7-BRIL N-terminus. **(B)** Aligned the curves in A shows protein homogeneity. **(C)** Diagram of SEC of different mutations at the UGT2B7-BRIL C-terminus. **(D)** Aligned the curves in **(C) (E)** Comparison of the SEC curves with the introduction of the N315A mutation. Flow rate: 0.5 ml/min. **(F)** Predicted structure of UGT2B7 using AlphaFold2 Protein Structure Database. The N-terminus and C-terminus are colored in orange and green and highlighted the side chain of Asn315.

Mutations have dual effects by increasing the protein stability and rendering the proteins in a specific conformation to alter the substrate selectivity (Kerdpin et al., 2009) (Xiong et al., 2008). After the introduction of BRIL, the yields of UGT2B7-BRIL were higher than those of the wild-type, but most of the proteins were inactive due to the aggregation during purification. Then we screened 17 mutations (Table 1). Compared with the wild-type, mutants of N68A and A71S at the N-terminus and mutants of G379D and D398N at the C-terminus improved the yields (Figures 3A,C. Supplementary Figure S3B). Aligned all results of SEC, mutants of Y268H and N315A showed better homogeneity, and mutants of N315A had higher yields (Figures 3B,D,E. Supplementary Figure S3D). It is assumed that the A71S, Y268H, and D398N substitutions could alter the pharmacodynamics or pharmacokinetics of many drugs, such as morphine (Sawyer et al., 2003), zidovudine (Court et al., 2003), and mycophenolic (Chung et al., 2008). We speculated that the changes in drug glucuronidation were possibly due to these mutations that increased the expression of UGT2B7 or reduced the amount of aggregated proteins. It is believed that the CTDs of UGTs are highly conserved and responsible for the UDPGA binding, and the distinct NTDs bind diverse substrates. Our results suggested that mutations in the conserved regions could have greater impacts on the protein stability. The other 11 mutations acted poorly in terms of yields and stability **(** Supplementary Figures S3B,D).

**TABLE 1:** Mutation screening for UGT2B7-BRIL.

Mutations	Original bases	Final bases	References
A71S	GCT	TCT	Yuan et al. (2015)
Y268H	TAT	CAT	Romero-Lorca et al. (2015)
D398N	GAT	AAT	
S15A	AGC	GCC	Miley et al. (2007)
H35A	CAT	GCT	
D151A	GAT	GCT	
T373V	ACT	GTT	
H374A	CAT	GCT	
N378S	AAT	GCT	
G379D	GGC	GAC	
N67A	AAC	GCC	Bateman et al. (2021)
N68A	AAC	GCC	Chen et al. (2009)
N315A	AAC	GCC	Nagaoka et al. (2012)
R259A	CGA	CTA	Nair et al. (2020)
Y33F	TAC	TTC	Chau et al. (2014)
Y33L	TAC	CTC	
N402A	AAC	GCC	

Protein glycosylation is considered as one of the major PTMs with significant effects on the protein folding, stability, and activity. UGT2B7 has been reported to be glycosylated at Asn68 and Asn315 (Nagaoka et al., 2012). When we mutated these two residues to alanine, both N68A and N315A mutations increased the protein expression, and N315A showed better homogeneity. We speculated that glycosylation might reduce the stability of the proteins during purification, probably due to the enormous function complexity and wide dynamic range distribution of the glycoproteins (Chen et al., 2009). Meanwhile, the structure of UGT2B7 predicted by the artificial intelligent AlphaFold2 Protein Structure Database (Jumper et al., 2021) showed that Asn315 is located in a highly flexible loop region (Figure 3F).

Based on our results of the insertion sites and mutations screening, we selected the full-length UGT2B7 (N315A)-BRIL construct with BRIL covalently attached to the C-terminus of UGT2B7 and the N315A mutation for further research (Figures 2A,c.)

#### 3.1.3 Negative-staining transmission electron microscopy

**FIGURE 4:**
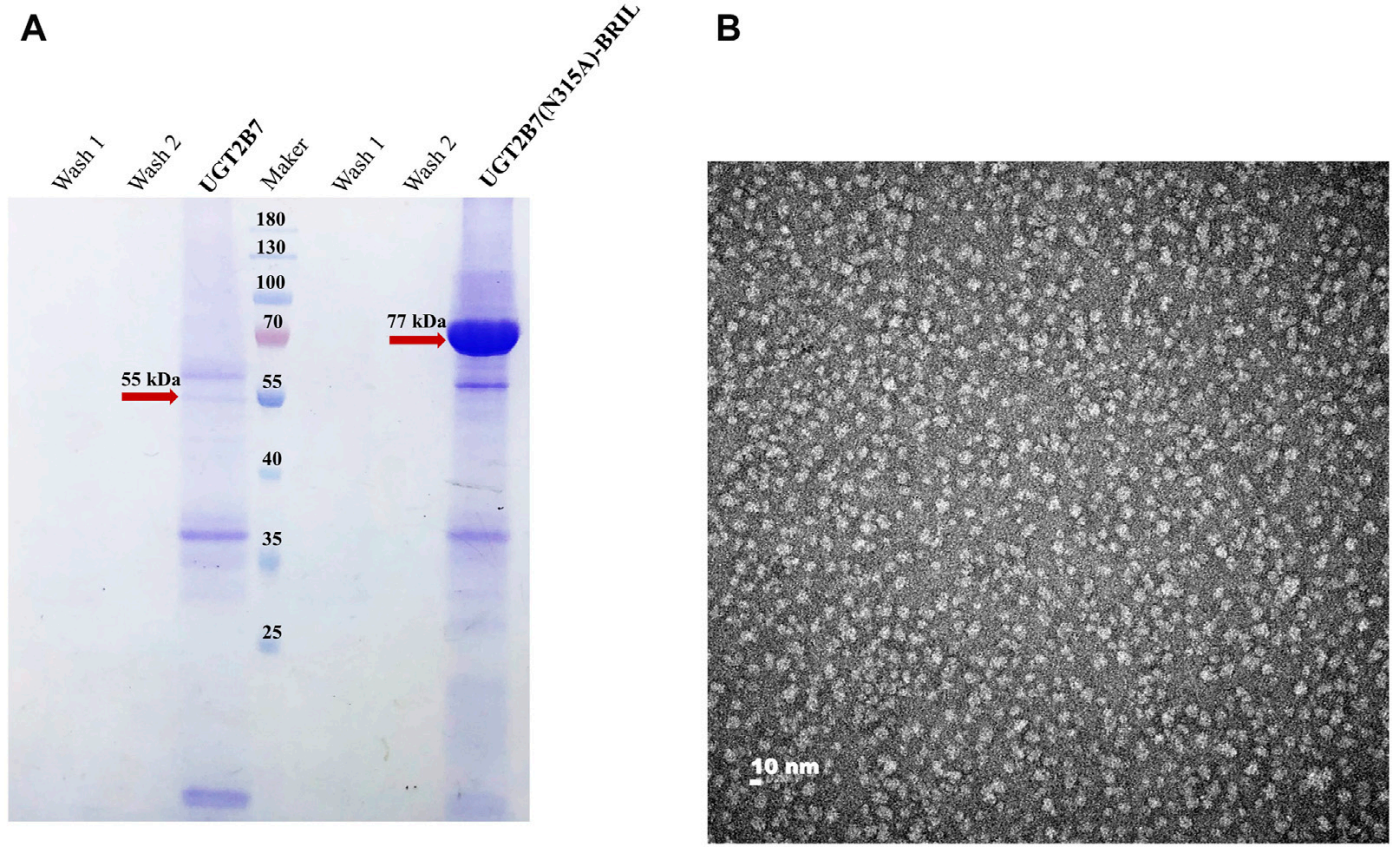
Characterization of UGT2B7(N315A)-BRIL. **(A)** Coomassie blue staining polyacrylamide gel electrophoresis of the wide type UGT2B7 (55 kDa) and UGT2B7(N315A)-BRIL (77 kDa). **(B)** Negative-staining TEM analysis of the UGT2B7(N315A)-BRIL protein particles at 180,000 × magnification.

With purification protocols described in the Materials and Methods, we obtained the full-length UGT2B7 (N315A)-BRIL proteins at >95% purity (Figure 4A). Electron micrographs of negative-staining UGT2B7 (N315A)-BRIL particles were uniformly distributed and showed particle sizes of 10 nm with oval shapes (Figure 4B). The high homogeneity of the particles indicated that UGT2B7 (N315A)-BRIL was stable and the purification protocols were rational for the biophysical characterization of UGT2B7.

### 3.2 Kinetics of zidovudine glucuronidation by recombinant UGT2B7

**FIGURE 5:**
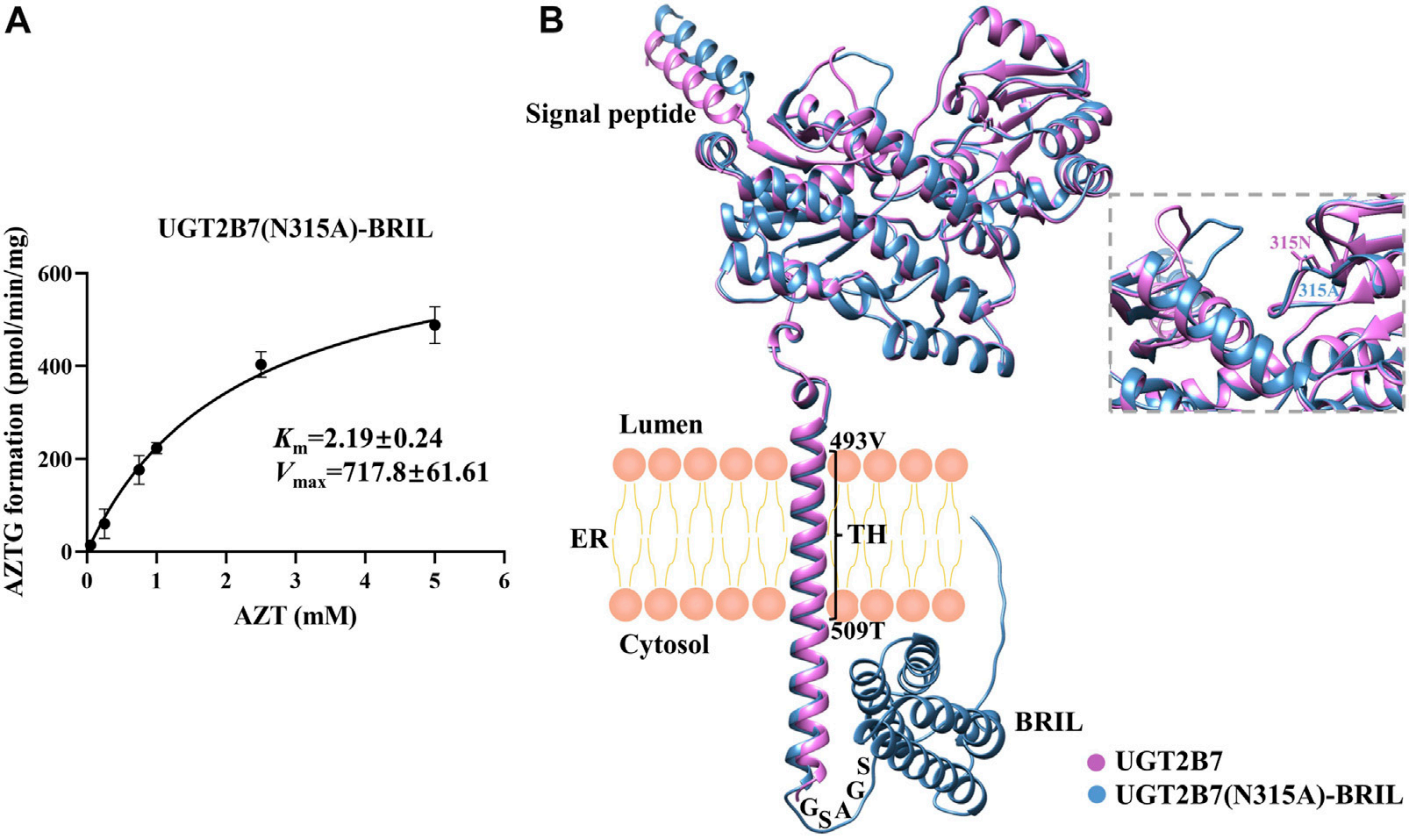
**(A)** Enzyme kinetics of AZTG formation of UGT2B7(N315A)-BRIL. Samples were analyzed by LC-MS/MS as described under Materials and Methods. AZT concentrations *versus* AZTG formation rates are shown. Glucuronidation rates are the mean ± standard deviation of three independent determinations. **(B)** Aligned three-dimensional structures of the UGT2B7 (pink) and UGT2B7(N315A)-BRIL (blue). The enlarged image highlighted the two side chains at the position 315. The numbers represent the residue positions of the wide-type UGT2B7 counted from the N-terminus. TH, transmembrane helix; ER, endoplasmic reticulum.

To analyze the catalytic activity of UGT2B7 (N315A)-BRIL against zidovudine, we investigated the kinetics of the formation of zidovudine-O-glucuronide (AZTG) by LC-MS/MS. The *V*_max_ showed 717.8 ± 61.61 pmol/min/mg and the *K*_m_ showed 2.19 ± 0.24 mM for AZT in insect cell homogenates (Figure 5A), which were consistent with the findings in the human liver microsome (HLM) (Horspool et al., 2020) (Girard-Bock et al., 2016). Meanwhile, we used the AlphaFold2 to predict the structures of UGT2B7 before and after modification (Figure 5B). It was found that the two key binding pockets at the luminal side might not be affected by the C-terminal fusion protein BRIL. It was likely that the flexible GSAGA linker helped BRIL flip freely in the cytoplasm, without changing the anchoring orientation of the single transmembrane helix.

### 3.3 Thermostability of recombinant UGT2B7 proteins

**FIGURE 6:**
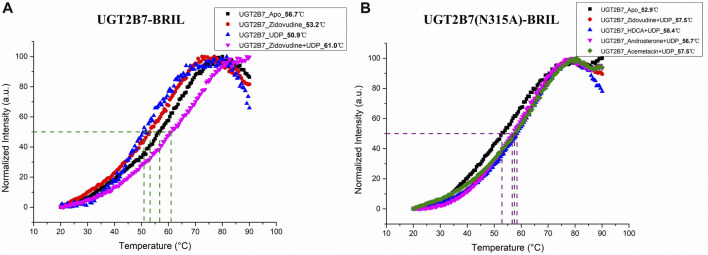
Thermostability of UGT2B7-BRIL. **(A)** the melting temperature (T_m_) of UGT2B7-BRIL in the presence of different compounds. **(B)** the T_m_ of UGT2B7(N315A)-BRIL in the presence of four substrates.

To assess the thermostability of the recombinant UGT2B7 proteins, we used the thiol-specific probes that fluoresced in response to the exposure of cysteines embedded within the helical bundle (Alexandrov et al., 2008). The melting temperature (T_m_, where half of the proteins are unfolded) of UGT2B7 in the absence of substrates was 56.7°C. Interestingly, the cofactor uridine 5′-diphosphate disodium salt (UDP, 50.9°C) or substrate (zidovudine, 53.2°C) made UGT2B7 less stable with lower T_m_ values (Figure 6A). However, the T_m_ value in the presence of both cofactor and substrate was increased (61.0°C). We speculated that the UGT2B7 proteins could be stabilized only by binding both cofactor and substrate, when the catalytic reaction had not undergone and high thermostability facilitated the glucuronidation reaction of the enzyme (Figure 6A). The mutation N315A showed no impact on the thermostability of the substrates binding to the enzyme, suggesting that the mutation might not be critical for the substrate bindings (Figure 6B).

### 3.4 Substrate binding affinity of UGT2B7

The substrates of UGTs exhibit a variety of chemical structures, with diverse properties of the functional groups that are glucuronidated. It is suggested that the substrate binding pocket is related to several regions of UGTs, including structurally conserved domains as well as loop regions. In contrast to assessing the UGT2B7 enzymatic activity using the human liver microsome (HLM) (Yang et al., 2017), we sought to directly analyze the binding affinity of the substrates to UGT2B7. The purified UGT2B7 (N315A)-BRIL proteins were immobilized on the SPR chip by coupling the 10 × His-tag at the UGT2B7 C-terminus to an anti-His tag antibody. The dissociation constants (*K*_D_) of the substrates and inhibitors to UGT2B7 were obtained by flowing these compounds across the surface of the CM7 sensor chip (Table 2).

**TABLE 2:** Calculated *K*_D_ and Chi^2^ for compounds binding to UGT2B7.

Compounds	*K*_D_ (μM)	Chi^2^ (RU^2^)
Zidovudine	3.67	0.952
Androsterone	8.82	0.257
Schisanhenol	17.3	0.067
Hesperetin	49.8	0.511
UDP	589	0.095

**FIGURE 7:**
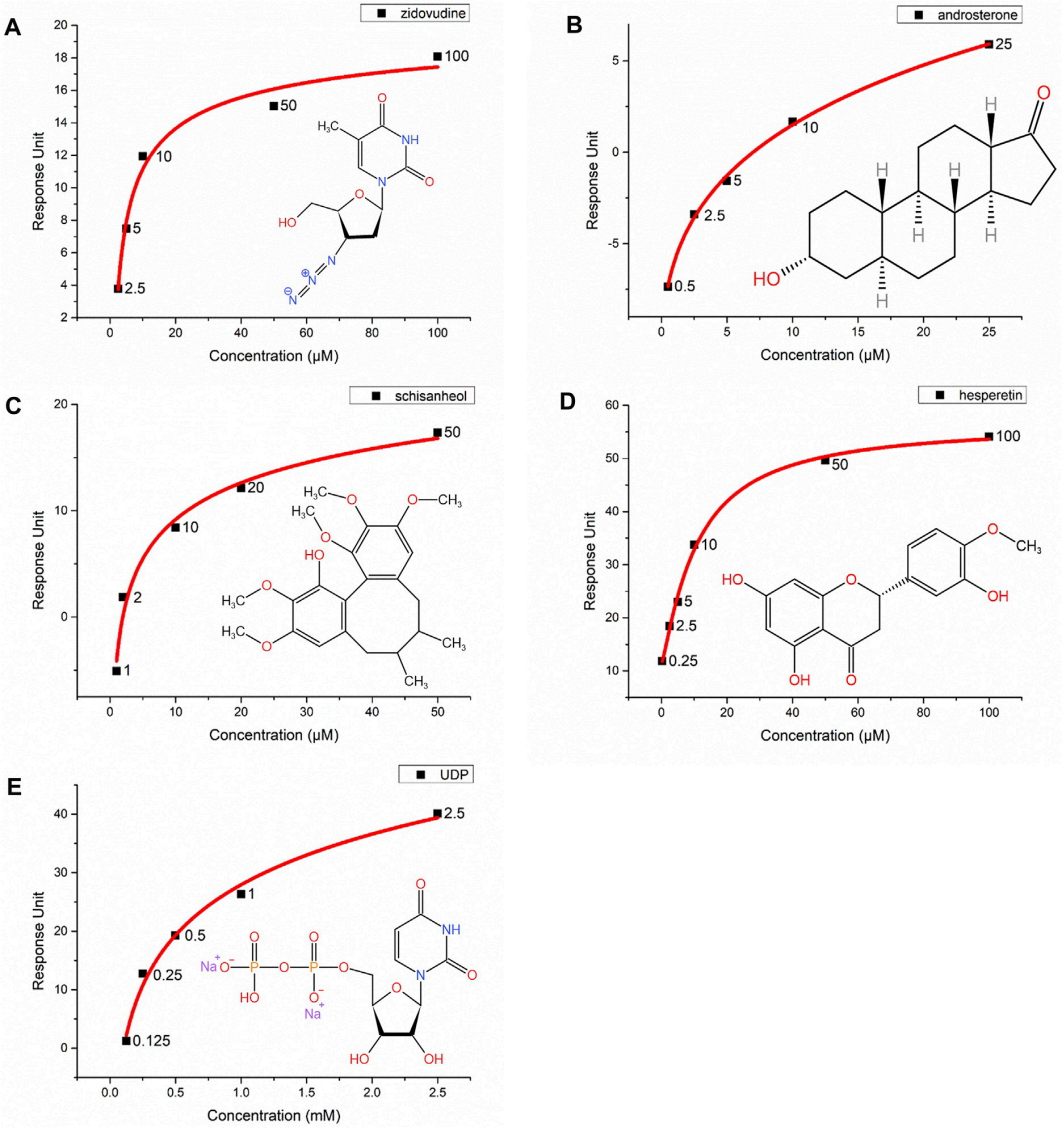
Interactions of five compounds with the UGT2B7(N315A)-BRIL proteins by surface plasmon resonance (SPR). Concentration-response curves of **(A)** zidovudine, **(B)** androsterone, **(C)** schisanhenol, **(D)** hesperetin, and **(E)** UDP. The *X*-axis represents the concentrations of substrates and *Y*-axis shows the response units from SPR. The chemical structures are marked at the right corner of each graph.

Zidovudine is often used as the probe substrate to determine the UGT2B7 activity (Badée et al., 2019). We measured the *K*_D_ value for zidovudine, at 3.67 μM, suggesting that zidovudine had a high affinity for UGT2B7 and our method was capable to study the substrate binding to UGT2B7 (Figure 7A). We also analyzed the *K*_D_ value of another classical substrate of UGT2B7, androsterone, at 8.82 μM, suggesting a lower affinity than that of zidovudine (Figure 7B).

Schisanhenol is a natural compound isolated from *Schisandra rubriflora* which exhibits effective inhibition against UGT2B7 (Song et al., 2015). Hesperetin, a natural flavonoid, with strong inhibition of UGT1A1, 1A3, and 1A9, and is a potent and broad-spectrum inhibitor against human UGTs (Liu et al., 2016). Our results suggested that the *K*_D_ value of schisanhenol was about three times higher than hesperetin. It was indicated that schisanhenol had a specific inhibitory effect on UGT2B7 (Figures 7C,D). We also analyzed the affinity of the glucuronidation products UDP for UGT2B7, in which its *K*_D_ value was much lower than those of the substrates or inhibitors, suggesting that the glucuronidation might not be inhibited by the product (Figure 7E). Our approach enabled rapid screening for enzyme inhibitors, which could help investigate the drug-drug interactions and guide the pre-clinical drug screening.

## 4 Discussion

Expression systems are important for the protein purification and characterization of UGTs. In this study, *Sf*9 insect cells were used to overexpress the proteins. Compared to the UGT2B7 nanodiscs (Cook et al., 2020) and the baculovirus-mammalian cell expression system (Miyauchi et al., 2022) to express the drug metabolic enzymes, our method is cost-effective and produces approximately 500 μg of membrane protein per liter of *Sf*9 cells, which is sufficient to explore the function, structure, and pharmacology of UGTs.

Two mutations in the signal peptide, L15A in UGT1A1 (Seppen et al., 1996) and P24T in UGT1A4 (Troberg and Finel, 2015), decrease the protein expression and glucuronidation rates. We investigated the role of the UGT2B7 signal peptide, using truncations and mutations, and found that the absence of the signal peptide prevented the protein from targeting the ER membrane and reduced the protein expression. We inserted BRIL to the C-terminus of UGT2B7, which increased the protein yields. In this way, the two binding pockets of UGT2B7 and BRIL were located on the opposite sides of ER, with minimal impact on the enzyme kinetics. At the same time, the GSAGS linker between the two proteins did not alter the orientation of UGT2B7 anchoring in the membrane.

UGTs are single transmembrane enzymes localized in the ER and their activities are usually reduced when isolated by detergents, suggesting that the enzyme activity is dependent on the phospholipids of the membrane (Radominska-Pandya et al., 2005). Previous studies show that the activities of eight human UGTs, fused with a 6 × His-tag at the C-terminus, are almost completely inhibited by 0.2% Triton X-100 (Kurkela et al., 2003). Meanwhile, the glucuronidation of morphine by the purified UGT1A10 proteins are not observed in the presence of 0.05% Triton X-100 (Kim et al., 2018). In our study, the glucuronidation of zidovudine by the purified UGT2B7 proteins in detergent LMNG/CHS was not observed, suggesting that the catalytic reaction of AZT relied on the phospholipid bilayer. Moreover, the T_m_ values changed when different substrates were used. Taken together, these results suggested that the absence of phospholipid bilayer might affect the glucuronidation of UGTs, rather than the substrates.

The human UGTs are involved in many clinically significant drug-drug interactions. Understanding the interaction mechanisms between different substrates and inhibitors are critical for predicting the drug-drug interactions, preventing the drug toxicity, and implementing the precision treatment (Yin et al., 2021). In recent years, researchers have investigated a variety of approaches for the enzyme kinetic analysis but rarely focused on the affinities of substrates. We used the SPR to explore the dissociation constants of the substrates with an attempt to reveal the substrate selectivity of UGTs. Our method enabled the measurement of *K*_D_ values for UGTs and the medium-throughput screening of enzyme inhibitors.

Drug metabolism has a significant impact on the pharmacokinetics, pharmacodynamics, toxicology, and other biomedical sciences. In the later stages of new drug development, the excellent performance of absorption, distribution, metabolism, and elimination (ADME) is one of the key factors in the pace of successfully developing effective drugs. The abnormal expression of various detoxification enzymes, such as UGTs, change the drug concentrations in the cells, resulting in chemical resistances that are difficult to reverse. Especially when the inherent and acquired drug glucuronidation is aberrant in the body, more research is needed to overcome this resistance. We established this novel method by the expression and purification of human full-length UGT2B7 proteins, which could be applied to the expression and purification of other enzymes in the UGTs family. The protein yields were sufficient for the three-dimensional structure determination of full-length human UGT2B7 in the future, and could aid the studies of pharmacokinetics and pharmacodynamics during the development of new drugs.

## Data Availability

The original contributions presented in the study are included in the article/Supplementary Materials, further inquiries can be directed to the corresponding authors.

## Abbreviations

AZT, zidovudine; AZTG, Zidovudine O-glucuronide; BRIL, apocytochrome *b*_*562*_RIL; CTD, C-terminal domain; ER, endoplasmic reticulum; HLM, human liver microsome; *K*_D_, dissociation constant; NTD, N-terminal domain; SEC, size-exclusion chromatography; SPR, surface plasmon resonance; TEM, transmission electron microscopy; TH, transmembrane helix; UDPGA, UDP-glucuronic acid; UGT, UDP-glucuronosyltransferase
