# Supplementary material for: Integrate thermostabilized fusion protein apocytochrome b 562 RIL and N-glycosylation mutations: A novel approach to heterologous expression of human UDP-glucuronosyltransferase (UGT) 2B7
Source: Front Pharmacol. 2022 Aug 12;13:965038. doi: 10.3389/fphar.2022.965038 (PMC9412022; doi:10.3389/fphar.2022.965038)
Supplement: Supplementary file 1 [file DataSheet1.docx]

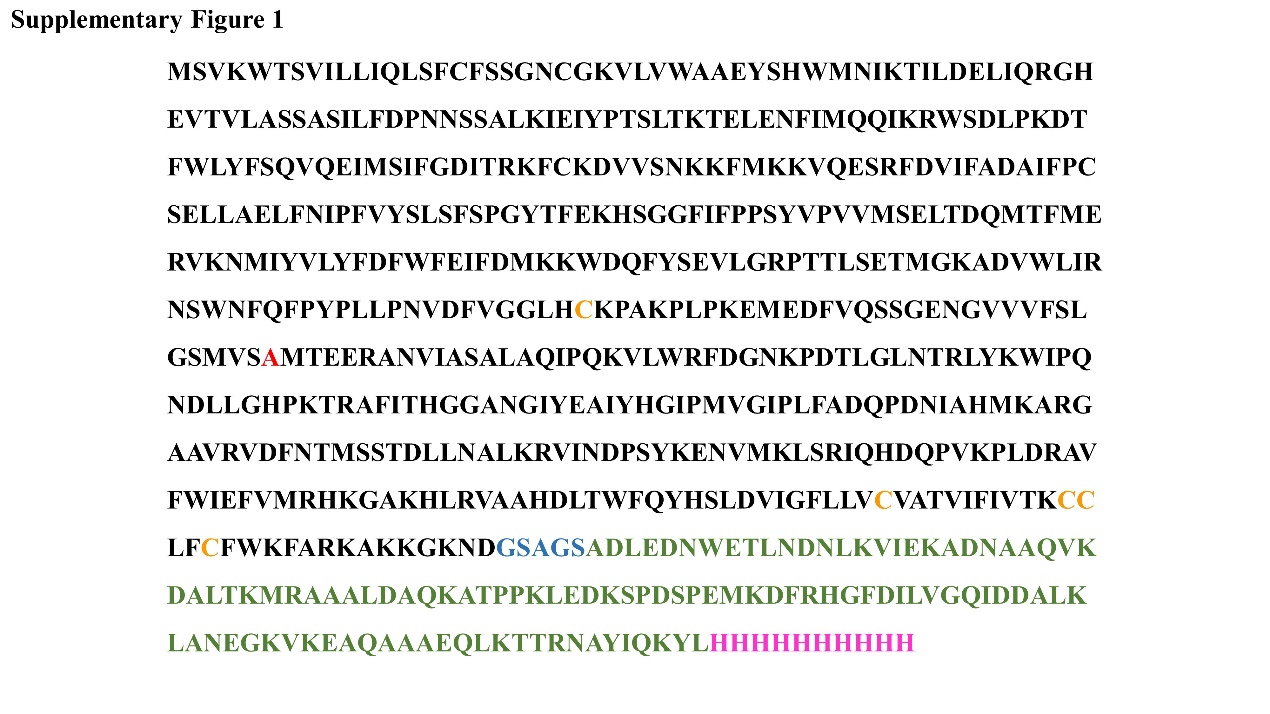


**Supplementary Figure 1.** The sequence of the engineered UGT2B7. The UGT2B7 (55 kDa) and fusion protein BRIL (12 kDa) are shown in black and green. N-Glycosylation mutation is shown in red, 5-residue linker GSAGS is colored in cyan and the 10 × His-tag is in magenta. The free Cys residues are shown in orange.


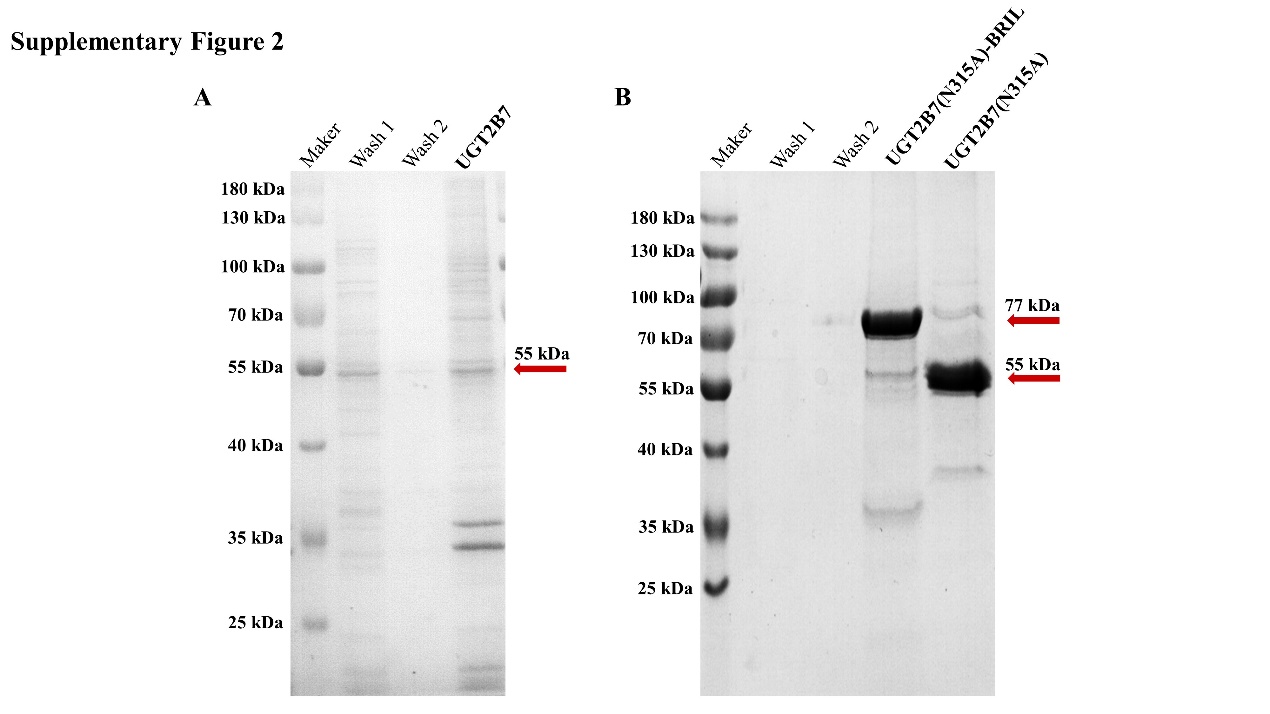


**Supplementary Figure 2.** To remove the fusion protein BRIL, we inserted a tobacco etch virus (TEV) cleavage site between the GSAGA linker and BRIL. The elute fractions that contained the purified UGT2B7(N315A)-BRIL proteins were concentrated to 0.5 mL using a 50 kDa molecular weight cutoff (MWCO) Vivaspin Turbo Ultrafiltration Unit and applied to the HP desalting column (GE Healthcare) equilibrated with the desalt buffer [20 mM HEPES (pH 7.5), 150 mM NaCl, 0.002% LMNG, and 0.0002% CHS] to remove the imidazole. The eluted proteins were treated overnight at 4℃ with 1 mg TEV protease (in-house made) to remove the C-terminal BRIL and 10 × His tag. The mixture was then incubated with Talon IMAC resin (TaKaRa) for 2 hours at 4℃, and cleaved UGT2B7(N315A) proteins were collected as the flow-through. Protein purity was assessed by SDS-PAGE. Coomassie blue staining polyacrylamide gel electrophoresis of **(A)** the wide type UGT2B7 proteins (55 kDa), and **(B)** UGT2B7(N315A)-BRIL (77 kDa). The last line in (B) showed the UGT2B7(N315A) (55 kDa) purity after the removal of BRIL.


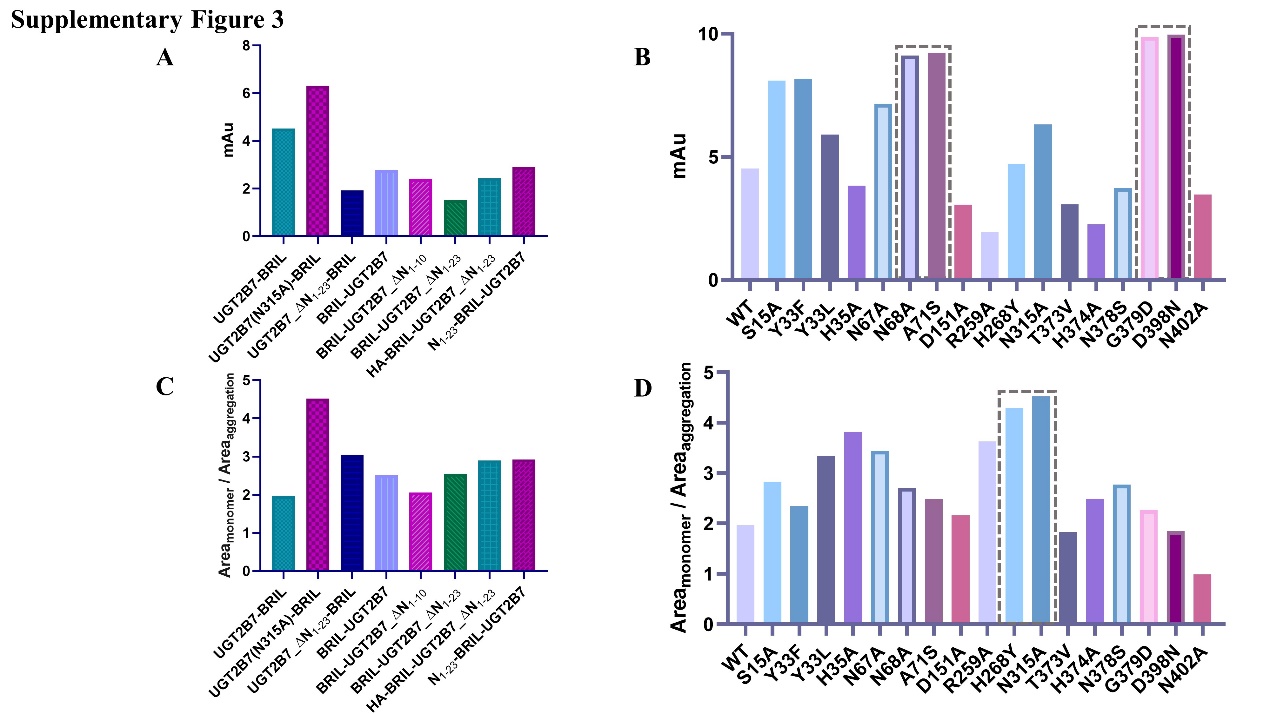


**Supplementary Figure 3.** Quantification of the SEC curves of the different constructs. **(A)** and **(B)** showed the yields, **(C)** and **(D)** showed the ratio of peak areas between monomer and aggregation proteins. **(A)** and **(C)**: Fused BRIL to the C-terminus of UGT2B7 with the N315A mutation significantly increased the yields and homogeneity of the full-length UGT2B7 proteins. **(B)** Compared with the wild-type, mutants of N68A and A71S at the N-terminus and mutants of G379D and D398N at the C-terminus improved the yields of UGT2B7 proteins. **(D)** Mutant of N315A showed better homogeneity.
